# The frequency of anti-phospholipid antibody syndrome in patients with premature coronary artery disease

**DOI:** 10.15171/jcvtr.2018.39

**Published:** 2018-12-13

**Authors:** Gholamhossein Alishiri, Maryam Moshkani Farahani, Ali Sadr, Mahmood Salesi, Mostafa Rahemi, Bahador Rezapoor

**Affiliations:** ^1^Baqiyatallah University of Medical Sciences, Tehran, Iran; ^2^Atherosclerosis Research Center, Baqiyatallah University of Medical Sciences, Tehran, Iran; ^3^Chemical Injuries Research Center, Systems Biology and Poisonings Institute, Baqiyatallah University of Medical Sciences, Tehran, Iran

**Keywords:** Anti-phospholipid Syndrome, Premature Coronary Artery Disease, Risk Factors

## Abstract

***Introduction:*** Coronary Artery Disease (CAD) is known as the major cause of morbidity and mortality in the world with a growing trend, especially in some developing countries. CAD commonly observed in elderly cases, however; recently it is usually found in young adults. In this study, we aimed to evaluate the prevalence of anti-phospholipid antibody syndrome (APS) in patients with premature CAD.

***Methods:*** The cross-sectional study was conducted in Baqiyatallah hospital from April 2012 to April 2016. Patients with premature CAD were included in the study. The data regarding the laboratory tests, echocardiography, and angiography were obtained from all cases.

***Results:*** Overall 133 eligible patients were included in the study. In the first set of the laboratory test, 18 patients were recognized to have APS (13.53%). The second confirmatory APA test was showing 3 of 18 patients were considered to have APS (2.25%).

***Conclusion:*** The results showed there is an association between the risk of developing Premature CAD and APS could potentially. The APS may have significant effects on the risk of coronary heart disease, especially in young adults.

## Introduction

Anti-phospholipid antibody syndrome (APS) is a systemic autoimmune disease.^1^ APS causes venous or arterial thrombosis and recurrent abortion.^2^ The laboratory findings in APS contains, persistently elevated levels of antibodies directed against membrane anionic phospholipids (anticardiolipin [aCL] antibody, anti-phosphatidylserine) or their associated plasma proteins, predominantly beta-2 glycoprotein I (apolipoprotein H); or evidence of a circulating anticoagulant.^3^

APS is known as the most common challenging hypercoagulable state. Approximately 65% of APS patients suffer from valvular heart disease and coronary artery disease (CAD). Also, APS may cause myocardial infarction (MI), pulmonary hypertension, intracardiac thrombus, and dilated cardiomyopathy.^4,5^

The vein and arterial thrombosis of APS antibodies have been reported as a cause of premature CAD.^6,7^ Studies on autoimmune diseases and cerebrovascular accident (CVA) have shown APS is more prevalent among young patients suffering from CVA.^8^

CADs occurring in women, less than 55 years old and in men, less than 45 years old is termed a young premature CAD.^9^ According to epidemiological studies based on angiographic finding the prevalence of premature CAD is reported about 10% worldwide.^10^

In this study, we aimed to evaluate the prevalence of APS in patients with premature CAD.

## Materials and Methods

The cross-sectional study was conducted in Baqiyatallah hospital from April 2012 to April 2016. All the patients with premature CAD were included. The inclusion criteria were; acute ST-elevation myocardial infarction (STE-MI), men less than 45 years old, women less than 55 years old, no addiction, no known chronic diseases (diabetes, hypertension, renal or hepatic failure).

The informed consent was obtained from all the patients. This study was approved by the ethics committee of Baqiyatallah University of medical sciences with registration number 1021.

The laboratory tests were done for all patients including; fasting blood sugar (FBS), 2 hours postprandial (2hpp), HbA1c, lipid profile, PT, PTT, anti-dsDNA, ANA, Anti B2 IgG & IgM, lupus anticoagulant, Anti-Car-IgG & IgM, insulin RIA and homocysteine. All laboratory tests were re-checked and repeated twice for determining specific and suspicious cases. The same results in both tests were described as definite whereas different results between the first test and the repeated test were considered as suspicious. The APS was confirmed if Anti B2 IgG & IgM, Lupus Anticoagulant, and anti-Car-IgG & IgM were positive after 12 weeks. The protein based laboratory tests were done by German-made ELISA kit.

The Ving Med B-Mode was used for measuring of echocardiography, and the angiography was performed in all patients by blinded physicians.

### Statistical analyses

The *t* test was used for quantitative comparison and the chi-square analysis was applied for qualitative variables. The *P* < 0.05 was chosen as the cutoff value of significance. All the calculations were statistically analyzed using the Statistical Package for Social Sciences (SPSS) software version 20.

## Results

In total, 133 eligible patients with premature CAD were selected and included in the study. In the first set of the laboratory test, 18 patients were recognized to have APS (13.53%). The second confirmatory APA test was showing 3 of 18 patients were considered to have APS (2.25%).

The mean (± standard deviation) age of patients was 46.94±6.32 years old and 60.9% (81) of the patients were female. The baseline characteristics of patients in the two studied groups (with and without APS) were described in Table 1. There were significant differences between the two groups in the frequency of history of deep vein thrombosis, aphthous ulcers and the use of oral contraceptive pills (OCP). Also, the average age of patients without APS was significantly older in comparison to APS patients, however the electrocardiogram (ECG) results were not statistically different between two groups: Axis (*P* = 0.14), ST elevation (*P* = 0.72), ST depression (*P* = 0.9) and T- inverting (*P* = 0.26).

**Table 1:** The baseline characteristics of patients in two studied groups

**Variables**	**Suspicious APS**	**Without APS**	***P*** ** value**
Age^a^	44.17 ± 6.02	47.37 ± 6.3	0.046
Gender (female)^b^	9 (50%)	72 (62.6%)	0.3
Body mass index^a^	21.24 ± 2.03	21.14 ± 1.81	0.83
Smoking^b^	15 (83.3%)	100 (87%)	0.67
Alcohol^b^	0 (0)	1 (0.9%)	0.69
Hypertension^b^	4 (22.2%)	39 (33.9%)	0.32
Diabetes^b^	4 (22.2%)	30 (26.1%)	0.72
Hyperlipidemia^b^	6 (33.3%)	23(20%)	0.2
Family history of CAD^b^	4 (22.2%)	15 (13%)	0.3
History of deep vein thrombosis^b^	3 (16.7%)	4 (3.5%)	0.02
Aphthous ulcers^b^	3 (16.7%)	6 (5.2%)	0.07
Oral contraceptive pills (OCP)^b^	4 (44.45%)	9 (12.5%)	0.05

^a^Independent *t* test; ^b^chi-square test.

The echocardiographic results showed no differences between two groups including mitral regurgitation (*P* = 0.21), tricuspid regurgitation (*P* = 0.19), pericardial effusion (*P* = 0.72) and aortic valve insufficiency (*P* = 0.4), although the differences between two groups in ventricular ejection fraction was significant (*P* = 0.05).

The angiographic results are presented in Table 2-4. The angiographic results indicated that the frequency of significant cut off in APS group patients was higher than other patients in right coronary artery (RCA) (OSTIUM). The angiographic results of left anterior descending (LAD) and left circumflex artery (LCx) showed no statistical differences between the two studied groups (Table 3 and Table 4). The laboratory results were demonstrated that these were significantly higher in the APS group in comparison to patients without APS with the exception of homocysteine (Table 5).

**Table 2:** The RCA angiographic findings of patients in two studied groups

**Angiographic findings**	**RCA (OSTIUM)**	**RCA (proximal)**	**RCA (Mid)**	**RCA (Distal)**
APS, No. (%)				
Normal	17 (94.4)	10 (55.6)	10 (55.6)	15 (83.3)
Non-significant	0 (0)	2 (11.1)	3 (16.7)	1 (5.6)
Significant	1 (5.6)	5 (27.8)	4 (22.2)	1 (5.6)
Cut-off	0 (0)	1 (5.6)	1 (5.6)	1 (5.6)
Non-APS, No. (%)				
Normal	11 (97.4)	83 (72.2)	75 (65.2)	89 (77.4)
Non-significant	3 (2.6)	10 (8.7)	17 (14.8)	12 (10.4)
Significant	0 (0)	13 (11.3)	17 (14.8)	10 (8.7)
Cut-off	0 (0)	9 (7.8)	6 (5.2)	4 (3.5)
*P* value*	0.03	0.26	0.84	0.84

*Chi-square test.

**Table 3:** The LAD angiographic findings of patients in two studied groups

**Angiographic findings**	**LAD (OSTIUM)**	**LAD (proximal)**	**LAD (Mid)**	**LAD (Distal)**
APS, No. (%)				
Normal	17 (94.4)	9 (50)	8 (44.4)	14 (77.8)
Non-significant	1 (5.6)	2 (11.1)	2 (11.1)	1 (5.6)
Significant	0 (0)	6 (33.3)	6 (33.3)	2 (11.1)
Cut-off	0 (0)	2 (11.1)	2 (11.1)	1 (5.6)
Non-APS, No. (%)				
Normal	104 (90.4)	6 (53.9)	6 (53.9)	91 (79.1)
Non-significant	6 (5.2)	16 (13.9)	16 (13.9)	8 (7)
Significant	3 (2.6)	31 (27)	31 (27)	13 (11.3)
Cut-off	2 (1.7)	6 (5.2)	6 (5.2)	3 (2.6)
*P* value*	0.84	0.62	0.68	0.91

*Chi-square test.

**Table 4:** The LCX angiographic findings of patients in two studied groups

**Angiographic findings**	**LCX (OSTIUM)**	**LCX (proximal)**	**LCX (Mid)**	**LCX (Distal)**
APS, No. (%)				
Normal	17 (94.4)	15 (83.3)	14 (77.8)	16 (88.9)
Non-significant	0 (0)	2 (11.1)	0 (0)	0 (0)
Significant	1 (5.6)	0 (0)	4 (22.2)	2 (11.1)
Cut-off	0 (0)	1 (5.6)	0 (0)	0 (0)
Non-APS, No. (%)				
Normal	113 (98.3)	91 (79.1)	92 (80)	91 (79.8)
Non-significant	1 (0.9)	8 (7)	8 (7)	6 (5.3)
Significant	1 (0.9)	14 (12.2)	12 (10.4)	13 (11.4)
Cut-off	0 (0)	2 (1.7)	3 (2.6)	4 (3.5)
*P* value*	0.29	0.31	0.31	0.62

*Chi-square test.

**Table 5:** The laboratory findings of two groups

Lab findings (positive)*	**APS**	**Non-APS**	***P*** value*
ANA, No. (%)	4 (22.2)	0 (0)	0.001
Anti-ds DNA, No. (%)	0 (0)	0 (0)	-
Anti B2 IgG, No. (%)	2 (11.1)	0 (0)	0.001
Anti B2 IgM, No. (%)	3 (6.7)	0 (0)	0.001
Lupus anticoagulant, No. (%)	8 (44.4)	0 (0)	0.001
Anti-Car-IgG, No. (%)	3 (16.7)	0 (0)	0.001
Anti-Car-IgM, No. (%)	3 (16.7)	0 (0)	0.001
Insulin RIA, No. (%)	6 (33.3)	16 (11.3)	0.02
Homocysteine, No. (%)	1 (5.6)	6 (5.2)	0.95

*Chi-square test.

## Discussion

APS as an auto-immune multisystem disorder is characterized by a high incidence of arterial and venous thrombosis. Cardiovascular manifestations also include valvular heart disease, ventricular thrombi and higher risk for CAD.^11^

Our results showed that the prevalence of APS in premature CAD patients was 2.25% in Baqiyatallah hospital (Tehran 2012-2016), however, the prevalence of APS in premature CAD patients reported about 3-10% in previous studies that is slightly higher but it has the adequate agreement with our reported results. The APS demonstrated in patients with repeated stent thrombosis in some case reports.^12-15^ The angiographic results of LAD and LCX showed no statistical differences between the two studied groups, but the RCA (OSTIUM) results were differences.

There were no differences between gender prevalence in the two studied groups. Although the previous studies on the epidemiology of APS demonstrated that APS is more prevalent in women than to men.^16^ The APS occurred more frequently in younger patients according to previous studies. The current studies indicate the average age is 44.17 in APS patients and 47.37 in without APS patients, and it is significantly different. In this study, all of the patients with confirmed APS were female, positive history of deep vein thrombosis and OCP medication. There was no significant difference in ECG results between the two groups.

The echocardiographic results showed no differences between the two groups; however, the average of EF was 49.44% and 44.17% for APS and non-APS group respectively.

The studies about APS and premature CAD are not frequent, Greco et al reported a study on 233 patients with considering gender, age, EKG, echocardiography that angiography results were very similar to our results.12 Most of the premature CAD patients with APS are female in rare case reports.^6,7,13,14^

In this study, most of the patients do not have CAD risk factors (diabetes, HTN, HPL, and smoking) and negative APS serologic tests, so seronegative APS must be considered. As a limitation of this study, the laboratory tests of phospholipid-binding plasma proteins and phospholipid-protein complexes are recommended for future studies. The studied sample size was in accordance with prevalence sample size formula however future multicenter studies with larger sample size could statistically improve test results.

## Conclusion

The results showed there is an association between the risk of developing Premature CAD and APS could potentially. The APS may have significant effects on the risk of coronary heart disease, especially in young adults.

## Ethical approval

This study was approved by the ethics committee of Baqiyatallah University of medical sciences with registration number 1021.

## Competing interests

None.
